# Genome-wide association study for morphological traits and resistance to *Peryonella pinodes* in the USDA pea single plant plus collection

**DOI:** 10.1093/g3journal/jkac168

**Published:** 2022-07-06

**Authors:** Lais B Martins, Peter Balint-Kurti, S Chris Reberg-Horton

**Affiliations:** Department of Crop and Soil Sciences, NC State University, Raleigh, NC 27607, USA; Department of Entomology and Plant Pathology, NC State University, Raleigh, NC 27607, USA; Plant Science Research Unit USDA-ARS, NC State University, Raleigh, NC 27695, USA; Department of Crop and Soil Sciences, NC State University, Raleigh, NC 27607, USA

**Keywords:** peas, *Pisum sativum*, GWAS, genome-wide association study, *Peryonella pinodes*, Ascochyta blight, USDA-PSPP

## Abstract

Peas (*Pisum sativum*) are the second most cultivated pulse crop in the world. They can serve as human food, fodder, and cover crop. The most serious foliar disease of pea cultivars worldwide is Ascochyta blight, which can be caused by several pathogens. Of these, *Peyronella pinodes* is the most aggressive and prevalent worldwide. Several traits, including resistance to *Peyronella pinodes*, stem diameter, internode length between nodes 2–3 and 5–6, and area of 7th leaf, were measured in 269 entries of the pea single plant plus collection. The heritability (H^2^) of the morphological traits was relatively high, while disease resistance had low heritability. Using 53,196 single-nucleotide polymorphism markers to perform a genome-wide association study to identify genomic loci associated with variation in all the traits measured, we identified 27 trait–locus associations, 5 of which were associated with more than 1 trait.

## Introduction

Dry peas (*Pisum sativum* L.) are the second most cultivated pulse crop in the world, coming after dry beans (FAOSTAT 2014; https://www.fao.org/faostat/en/). The United States is the 4th largest producer and 3rd-largest exporter globally (Rawal and Navarro 2019). Peas can serve as human food, fodder, and cover crop. They fix biological nitrogen and can add up to 146 kg N ha^−1^ (Parr *et al.* 2011). Pea production can be affected by various abiotic and biotic stresses (Diego Rubiales *et al.* 2015). The most serious foliar disease of pea cultivars worldwide is Ascochyta blight (Bernard Tivoli *et al.* 2007), which can cause up to 75% yield loss under conditions that are favorable for the disease (Bretag *et al.* 2006). Ascochyta blight is a disease complex caused by several related fungal pathogens individually or in combination (Bernard Tivoli *et al.* 2007). In North America, the species associated with the disease are *Didymella pisi*, *Peyronellaea pinodella*, and *Peyronellaea pinodes* (Skoglund *et al.* 2011; Owati *et al.* 2020); the last of these being the most aggressive and prevalent worldwide (Rubiales *et al.* 2019; Xue *et al.* 1997; Bretag *et al.* 1995; Tivoli *et al.* 1996). Pathogens are very hard to discriminate based on the symptoms they cause, as all of them cause black to brown spots or lesions on leaves, stems, and pods (Skoglund *et al.* 2011). However they can be distinguished based on other characteristics such as spore characteristics, appearance on culture plates, and toxin production (Owati *et al.* 2020).

Ascochyta blight can be controlled with agronomic practices (Bretag *et al.* 2006; Liu *et al.* 2016). The use of clean and treated seed will prevent seed-born inoculum. Crop rotation, destruction of infected pea trash, and adjustment to the planting date will decrease the odds of soil and air-born inoculum infestation. To remediate losses, fungicides can also be applied but this results in increased input costs for the farmer. A more sustainable approach is to breed for resistance to the pathogen. So far, low levels of polygenic resistance to *P. pinodes* have been identified in *Pisum sativum* (Parihar *et al.* 2020), and high levels of resistance have been reported in wild peas (Fondevilla *et al.* 2008; Wroth 1998; Ambuj Bhushan Jha *et al.* 2012; Jha *et al.* 2017). Disease resistance has been associated with lower plant growth in many plant systems (Brown and Rant 2013). Although the causes of the tradeoff between growth and defense are not entirely known, they may be based partially on antagonistic signaling pathways and the metabolic costs of the defense response (Karasov *et al.* 2017). Since plant vigor is also paramount for a breeding program, it is important to know if disease resistance in a population is associated with undesired traits, such as hindered plant growth.

Although peas were the original system used for genetic studies (Ellis *et al.* 2011), the reference genome was only published in 2019 (Kreplak *et al.* 2019). Pea (2*n* = 14) has a large, highly repetitive genome, of 4.45 Gb, almost twice as large as maize (2*n* = 20, 2.4 Gb) and almost 4 times that of soybean (2*n* = 20, 1.15 Gb). To preserve genetic diversity and aid in genomic-assisted breeding, the USDA assembled the pea single plant plus collection (PSPPC) (Holdsworth *et al.* 2017), which comprises 431 morphologically, geographically, and taxonomically diverse *P. sativum* accessions. Detailed genotypic data are publicly available for the PSPPC, along with 25 accessions of *Pisum fulvum*, a wild relative of peas (Holdsworth *et al.* 2017). Genome-wide association studies (GWAS) aim to connect underlining genetics to traits of interest, and requires a panel with diverse genotypes (Korte and Farlow 2013), the PSPPC is ideal for such studies.

All the published quantitative trait loci (QTL) mapping studies for resistance to *P. pinodes* have used biparental populations which can only detect effects at loci for which the parents possess functionally distinct alleles. GWAS, in contrast, has the potential to assess the relative effects of multiple alleles at each locus.

This study describes the first GWAS for resistance to *P. pinode* as well as several morphological traits.

## Materials and methods

### Plant materials

Two hundred and sixty-nine *P. sativum* lines from the PSPPC collection were planted in a randomized complete block design in a growth chamber at the Phytotron at North Carolina State University, Raleigh, NC, USA. Cultivars Radley and Solara were used as resistant and susceptible checks, respectively. Due to space limitations, only 1 replicate was planted at a time. The experiment was run 7 times, i.e. in 7 replications. For each replication, 3 seeds per entry were planted in a 7.5-cm (225 ml) Styrofoam cup and thinned to 1 plant per plot before inoculation. The substrate used was 50% Sun Gro Propagation Growing Mix (Canadian Sphagnum peat moss 50–65%, vermiculite, dolomitic lime, 0.0001% silicon dioxide), and 50% cement sand. The cups were organized in metal trays holding 5 cups, and 4 metal traits were in each rolling cart. The chamber was kept at 21°C with 12 hr of light, and air humidity fluctuated between 50% and 100%. Plants were not inoculated with rhizobium but were fertilized 3 times a week when watered with a standard nutrient solution in the growth chambers containing nitrogen, phosphorus, potassium, and other micronutrients (https://phytotron.ncsu.edu/general-information/). As the plants grew, they were trellised using thin bamboo stakes.

### Pathogen and inoculation

Six isolates of *P. pinodes* originating from different countries were obtained from the USDA collection kept in Washington (Supplementary Table 1). The pathogen was grown on potato dextrose agar plates for 12–15 days under 12 hr of light and 21°C. Pycnidia were harvested from the plates by gently flooding the plates with a solution of 0.12% Tween 20 in water followed by gentle brushing of the surface of the plate. The pycnidia were counted and the final inoculum concentration was adjusted to 5 × 10^5^ per ml in the same 0.12% Tween 20 solution (Fondevilla *et al.* 2005). The inoculum was prepared immediately before inoculation and was kept on ice to prevent premature spore germination. The entire procedure, from spore isolation to inoculation of the whole population took about ∼3 hr.

Plants were inoculated 10–13 days after planting when most of the plants were at the 3–4 expanded leaf stage. In the growth chamber, whole individual plants, and both sides of the leaves, were sprayed with inoculum solution using an airbrush until dripping, using approximately 1 ml of inoculum per plant. After inoculation, each metal tray containing 5 plants was covered in a clear plastic bag and tied to create high humidity conditions favorable to pathogen growth. After 96 hr, the bags were removed.

### Phenotyping

The first 3 leaves were individually scored on a 0–6 scale developed by Schoeny *et al.* (1996), in which 0 corresponds to no symptoms and 6 to necrotic lesions in 75–100% of leaf area. Plants were scored 3–4 times over 10 days. On the final scoring, the total number of nodes of each plant was recorded. On the day after the final score, plants were cut at the base and imaged using an Epson Perfection V500 Photo Scanner. The 7th fully expanded leaf, or the 6th in case there was not a 7th, was removed and laid flat for the scanning. Images were obtained for 5 replicates and processed using the software ImageJ (Rasband 1997). Traits measured were internode lengths between nodes 2–3 and 5–6, stem diameter between nodes 5 and 6, and area of 7th or 6th leaf. These traits will be referenced throughout the article as internode 2–3, internode 5–6, diameter, and leaf area, respectively.

### Statistical analysis

The distribution of each replicate for each trait was visualized using the package ggplot2 (Wickham 2016) in R studio. Outliers were defined as observations outside 1.5 times the interquartile range above the upper quartile and below the lower quartile and were excluded. Broad sense heritability (H^2^) was calculated for each trait by dividing the variance due to genotype by the total variance for the phenotype, which was the sum of variance due to genotype, variance due to the interaction of genotype by replication, and variance due to error (*V*_G_/(*V*_G_ + *V*_GR_ + *V*_e_)).

A single disease score per plant for each date was calculated as an average of the score of 3 leaves scored. For each replication, the standardized area under disease progress curve (sAUDPC) was calculated by averaging the value of 2 consecutive ratings and multiplying by the number of days between the ratings. This was done for each set of consecutive ratings. Values were then summed over the intervals and divided by the total number of days between the first and last evaluations (Campbell and Madden 1990). This calculation means that the sAUDPC scores are on the same scale as the original 1–6 scoring scale.

$$\mathrm{sAUDPC}=\frac{\sum_{i=1}^{n}\left[\frac{y_{i}+y_{i+1}}{2}\right]\left(t_{i+1}-t_{i}\right)\;}{t_{n}}$$

where *y_i_* is the disease score on the *i*th date, *t_i_* is the number of days since the first disease evaluation, and *t_n_* is the total number of days between first and last scoring.

Best linear unbiased predictors (BLUPs) were calculated for each entry and trait. The ASReml package (Butler *et al.* 2018) in R studio was used to calculate BLUPS for sAUDPC and the lmer4 (Bates *et al.* 2015) was used to calculate BLUPS for internode 2–3, internode 5–6, diameter, and leaf area. To calculate BLUPs for sAUDPCs the following model was fit

$$y_{\mathrm{ijkmn}p}=\mu +G_{i}+R_{j}+GR_{ij}+SR_{kj}+TCR_{\mathrm{mnj}}+NR_{pj}+e_{\mathrm{jkmnp}}$$

where *y* is the response variable sAUDPC, and the random effects are: *G_i_* is the effect of genotype, *R_j_* is the effect of replicate, *GR_ij_* is the interaction effect of genotype and replicate, *SR_kt_* is the effect of side of the chamber nested in replicate, *TCR_mnj_* is the effect of the inoculation unit of tray nested in cart and replicate, *NR_pj_* is the effect of the number of nodes nested in replicate.

A simpler model was used to calculate BLUPS for internode 2–3, internode 5–6, diameter, and leaf area

$$y_{ij}=\;\mu +G_{i}+R_{j}+GR_{ij}+e_{ij}$$

where *y* is the response variable of each trait, *G_i_* is the effect of genotype, *R_j_* is the effect of replicate, and *GR_ij_* is the interaction effect of genotype and replicate.

Processed GBS genotype data aligned with the reference genome was obtained from Powers *et al.* (2021). The data were then filtered for minor allele frequency (MAF) (retaining alleles with >0.05 frequency) and heterozygosity (retaining lines with lower than 20% heterozygosity) using the software TASSEL (Bradbury *et al.* 2007). The GAPIT (Lipka *et al.* 2012) package in R was used to perform GWAS on each trait, using the Bayesian information and linkage disequilibrium iteratively nested keyway (BLINK) model. This model was selected because it has higher statistical power than the model fixed and random model circulating probability unification (FarmCPU). BLINK does not assume that causal genes are distributed evenly across the genome, as FarmCPU does, leading to fewer false positives and exclusion of causal genes (Huang *et al.* 2019). While FarmCPU divides the genome in equal sized bins to find the most significant marker in each bin, BLINK groups SNPs based on linkage disequilibrium regardless of how physically close they are, allowing the discovery of clusters. The BLINK models were fit as:

$$y=s_{i}+S+e$$

where *y* is a vector of phenotypes; *s_i_* is a testing marker; *S* is a pseudo quantitative trait nucleotide; and *e* is the unobserved vector of residuals.

False discovery rate (FDR) adjusted *P*-values, with a threshold of 0.05, was calculated for all single nuclear polymorphisms (SNPs) using GAPIT (Lipka *et al.* 2012). The GAPIT (Lipka *et al.* 2012) package was also used to select the optimal number of principal components (PC) included in the model using a BIC; PC reflects the degree of population structure that should be accounted for in the model. The results indicated that no PCs were necessary in the model.

### Candidate genes

Genes within 1 Mb of highly associated SNPs, as identified using the *P. sativum* v1a genome (https://www.pulsedb.org/jbrowses), were considered as candidate genes.

## Results and discussion

For this experiment, 269 of the 431 lines that comprise the PSPPC were available for use. The lines were evaluated in controlled conditions for resistance to *P. pinodes*, and for 4 other morphological traits: 7^th^ leaf area, internode length between nodes 2 and 3, internode length between internodes 5 and 6, stem diameter between nodes 5 and 6. The experiment was run in 7 replications in a complete block design, with 1 full replicate per run. sAUDPC correlations between replicates were highly significant and ranged from *r *=* *0.16 to 0.35 (*P*-values <0.01) (Supplementary Table 2) and the broad-sense heritability for this trait was 0.3 (Table 1). Replicate was the largest source of variance for sAUDPC (Supplementary Table 3). The most likely causes of variation between replicates, and for the consequent moderate correlations and heritability we observed for sAUDPC, were variation in the inoculum, which was prepared fresh for each run. Replicate was also a substantial source of variance for most of the physiological traits measured (Supplementary Table 3). While growth chamber conditions were constant across replicates, variation in plant growth caused by factors such as variations in seed quality, germination speed, planting depth and local microclimates, may have also been a factor. Variation in plant growth was also a likely cause of variation in disease symptom measurements.

**Table 1.** Broad sense heritabilities of traits.

Trait	H_2_
Disease resistance	0.30
Internode 2–3	0.70
Internode 5–6	0.78
Leaf area	0.70
Stem diameter	0.72

**Fig. 1.**
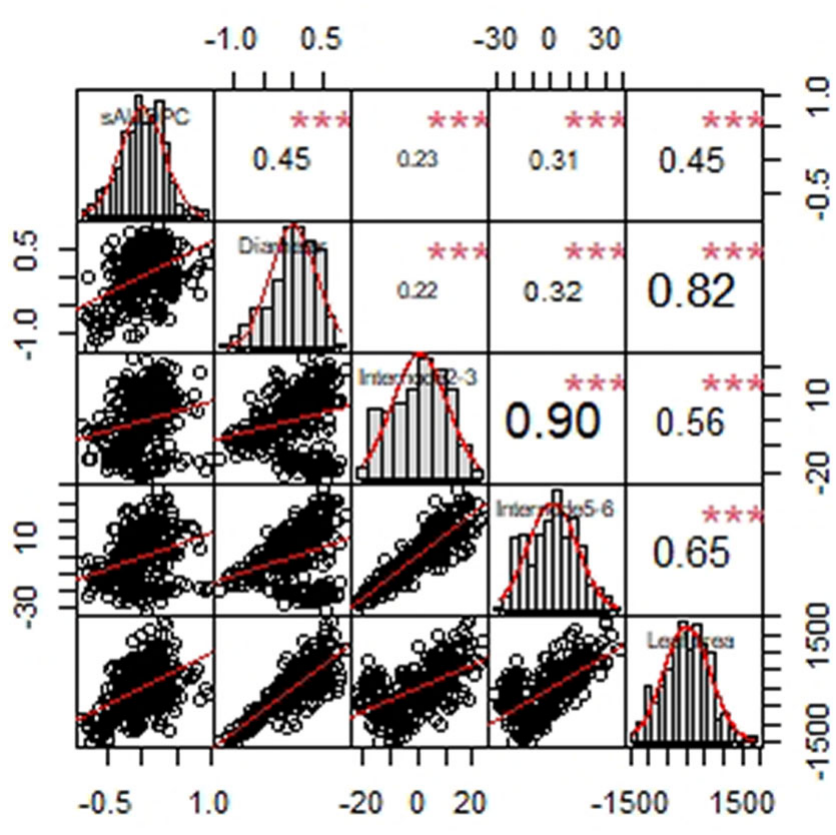
Distribution, correlations, and scatter plots of each trait BLUPs. Line for the best fit of data is displayed. *** correspond to *P*-value <0.001.

The 4 morphological traits (leaf area, diameter, internode 2–3, internode 5–6) were measured in 5 replicates. Each morphological trait had a higher correlation between replicates than sAUDPC, ranging from *r *=* *0.58 to 0.84 (*P*-values <0.01 for each trait and Supplementary Table 2), and broad-sense heritability ranging from 0.7 to 0.78 (Table 1). BLUPs were calculated for every trait and the largest source of variation was always entry (Supplementary Table 3). Correlations between BLUPs for all traits, including sAUDPC, ranged from 0.22 to 0.9 and were significant at a *P*-value < 0.01 (Fig. 1). Since the morphological traits are all parameters of plant growth, it was expected that they would be highly correlated. Disease resistance was moderately correlated with all the growth traits, suggesting that lower growth was associated with higher disease resistance. Associations between lower plant growth and higher disease resistance have been observed frequently in a number of plant systems (Brown and Rant 2013). The basis of the so-called “growth-defense tradeoff” is not entirely understood but may be based partially on antagonistic signaling pathways and the metabolic costs of the defense response (Karasov *et al.* 2017).

**Fig. 2.**
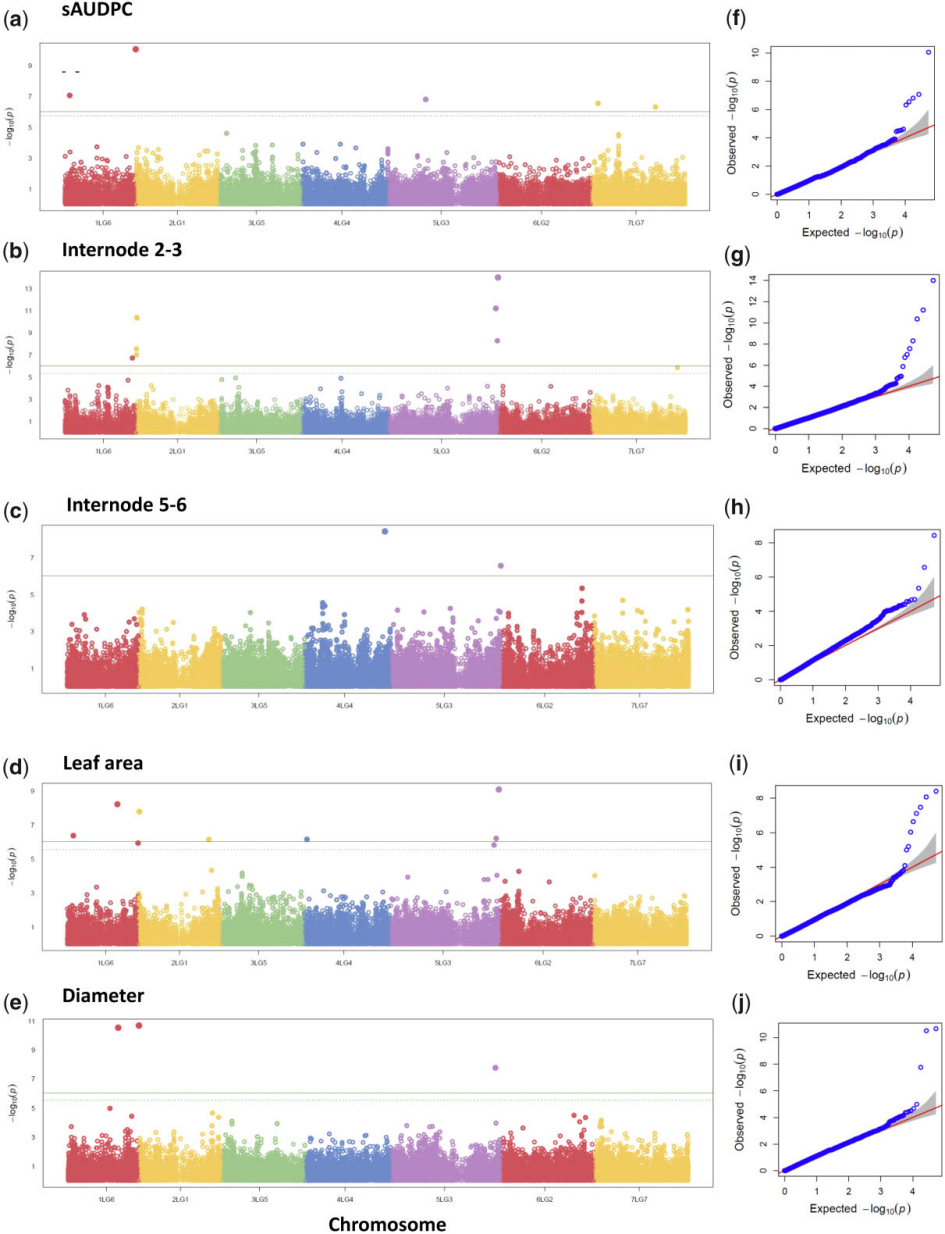
Manhattan plots and the corresponding Q–Q plots for the traits indicated. a, e) **s**AUDPC for *P. pinodes*. b, f) Internode length between nodes 2 and 3. c, g) Internode length between nodes 5 and 6. d, h) Area of 6th leaf. e, i) Stem diameter between nodes 5 and 6. In the Manhattan plots, the −log_10_*P*-values adjusted for FDR (*y*-axis) are plotted against the position of each chromosome (*x*-axis), each circle represents an SNP, solid green line represents FDR adjusted *P*-value threshold of 0.005 and dotted line threshold of 0.05.

After filtering 319,141 SNPs for MAF (>0.05) and heterozygosity (<20%), 53,196 SNPs were used for GWAS. Q–Q plots for each GWAS suggested that models had a good fit and that reliable SNP–trait associations were identified (Fig. 2). In total, 27 significant trait-locus associations were identified across all 5 traits (Fig. 2 and Table 2). The high phenotypic correlations between some of the growth-related traits, especially leaf area and stem diameter and between internodes 2–3 and internodes 5–6 were reflected in shared genetic control of the traits. Five SNPs were associated with more than 1 trait; 2 SNPs (S1LG6_261934321, S5LG3_544595701) were associated with both stem diameter and leaf area, 1 SNP was associated with internode 2–3 (S5LG3_569851018) and leaf area, and 1 SNP (S5LG3_572900348) was associated with internode 2–3 and internode 5–6 (Table 2). One SNP (S1LG6_369964198) on chr1 (LG6) was associated with 3 traits, sAUDPC, leaf area, and stem diameter. All the SNPs that were significant for more than 1 trait had the minor allele phenotype related to a smaller plant; shorter internode, smaller leaf, thinner stem, and less disease. After calculating the physical distance between significant SNPs, only SNPs S2LG1_353493 and S2LG1_528924 were closer than 1 Mb, both were associated with internode 2–3, but 1 had the minor allele trait as longer internode while the other longer internode (Table 2). In Supplementary Table 4, we list the 415 predicted genes within 500-kb flanking significant SNPs. This constitutes a very preliminary list of candidate genes that might be involved in controlling these traits.

**Table 2.** Significant SNPs for stem diameter, leaf area, internode 2–3, internode 5–6, and sAUDPC.

SNP^*a*^	Chr^*b*^	SNP_Position	FDR adj *P*-val^*c*^	MAF^*d*^	Trait	Alleles^*e*^	MA trait^*f*^
S1LG6_26051507	1LG6	26051507	4.57E−04	0.25	sAUDPC	C/T	Resistance
S1LG6_31928165	1LG6	31928165	5.28E−03	0.27	Leaf area	T/C	Smaller leaf
S1LG6_261934321	1LG6	261934321	6.00E−04	0.19	Leaf area	C/T	Smaller leaf
S1LG6_261934321	1LG6	261934321	1.51E−06	0.19	Stem diameter	C/T	Thinner stem
S1LG6_351220787	1LG6	351220787	1.40E−03	0.09	Internode 2–3	T/C	Shorter internode
S1LG6_369964198	1LG6	369964198	7.35E−03	0.20	Leaf area	A/C	Smaller leaf
S1LG6_369964198	1LG6	369964198	1.00E−02	0.20	sAUDPC	A/C	Resistance
S1LG6_369964198	1LG6	369964198	5.00E−08	0.19	Stem diameter	A/C	Thinner stem
S2LG1_353493	2LG1	353493	8.64E−04	0.07	Internode 2–3	A/C	Longer internode
S2LG1_528924	2LG1	528924	2.97E−04	0.44	Internode 2–3	A/T	Ahorter internode
S2LG1_3521266	2LG1	3521266	7.60E−07	0.48	Internode 2–3	A/T	Shorter internode
S2LG1_4685463	2LG1	4685463	2.11E−04	0.49	Leaf area	C/T	Larger leaf
S2LG1_367524526	2LG1	367524526	1.02E−03	0.16	Leaf area	G/A	larger leaf
S4LG4_14403384	4LG4	14403384	5.28E−03	0.08	Leaf area	T/C	Smaller leaf
S4LG4_416335752	4LG4	416335752	1.95E−04	0.14	Internode 5–6	A/T	Shorter internode
S5LG3_198269966	5LG3	198269966	4.86E−03	0.11	sAUDPC	C/T	Resistance
S5LG3_544595701	5LG3	544595701	8.25E−03	0.20	Leaf area	G/A	Smaller leaf
S5LG3_544595701	5LG3	544595701	1.35E−03	0.20	Stem diameter	G/A	Thinner stem
S5LG3_555981910	5LG3	555981910	5.28E−03	0.46	Leaf area	G/C	Larger leaf
S5LG3_561689517	5LG3	561689517	1.62E−07	0.39	Internode 2–3	G/A	Shorter internode
S5LG3_569851018	5LG3	569851018	6.77E−05	0.10	Internode 2–3	T/G	Shorter internode
S5LG3_569851018	5LG3	569851018	2.28E−04	0.10	Leaf area	T/G	Smaller leaf
S5LG3_572900348	5LG3	572900348	5.21E−10	0.26	Internode 2–3	A/G	Shorter internode
S5LG3_572900348	5LG3	572900348	7.14E−03	0.26	Internode 5–6	A/G	Shorter internode
S7LG7_37540311	7LG7	37540311	1.10E−03	0.29	sAUDPC	T/C	Resistance
S7LG7_336950420	7LG7	336950420	6.78E−04	0.35	sAUDPC	C/T	Resistance
S7LG7_449022566	7LG7	449022566	9.02E−03	0.12	Internode 2–3	A/T	Shorter internode

a SNP names.

b Chromosome and LG.

c SNP FDR-adjusted *P*-value.

d Minor allele frequency.

e Alleles associated with SNP (minor allele comes first).

f Effect associated with minor allele (MA trait).

The reference genome was published only 3 years ago (Kreplak *et al.* 2019) and many previous QTL and SNPs positions have been reported by linkage group (LG) instead of chromosome (chr). Here we will refer to the SNP position according to the chromosome and its associated LG reported in the reference genome (Kreplak *et al.* 2019). We used the BLAST tool available at PulseDB website (https://www.pulsedb.org/blast) to find the location on the reference genome of previously identified significant markers and proteins. Past studies conducted both in the field and in controlled conditions, found QTL associated with resistance to *P. pinodes* on multiple chromosomes (Fondevilla, Küster, *et al.* 2011; Prioul *et al.* 2004; Prioul-Gervais *et al.* 2007; S. Fondevilla *et al.* 2008, 2018; Carrillo *et al.* 2013, 2014; Fondevilla, Almeida, *et al.* 2011; Timmerman-Vaughan *et al.* 2004, 2002; Castillejo *et al.* 2020; Jha *et al.* 2017). Through the use of proteomics and gene expression studies, some candidate genes have been reported (Fondevilla *et al.* 2018; Castillejo *et al.* 2020), none of those were found to be within 500 kb flanking of the significant SNPs found in the current study. One reason for this may have been that previous studies relied on biparental populations, which will have fewer segregating alleles than in a diversity panel, and those alleles could be different than the ones segregating in this population. Experimental conditions and pathogen strain used can also be a cause of different results, most of the previous studies were phenotyped in the field, while we used controlled conditions. Little is known about the difference between the strains used in the present and past studies and the existence of *P. pinodes* pathotypes have been reported (Khan *et al.* 2013). If the strains belong to different pathotypes, difference in resistance could be observed. Furthermore, past studies utilized very few markers in comparison with the present study, which can result in a lower resolution for the QTL positioning.

The *Le* gene, described by Mendel (Ellis *et al.* 2011), was the first gene found to be associated with internode length in pea. The gene was mapped on chr5(LG3) (Psat5g299720.1, position: chr5LG3:567365719.567368443), and shown to affect gibberellin (GA) biosynthesis (Lester *et al.* 1997). GA affects many aspects of plant growth, including stem elongation and leaf expansion (Achard and Genschik 2009). Although *Le* plays a significant role in stem length, different studies have found multiple regions of the genome associated with internode length, suggesting that the trait is more complex than previously suggested by Mendel’s studies (Gali *et al.* 2019; Tafesse *et al.* 2020). Today we know that there are at least 3 more genes involved in the production of GA (Reid and Potts 1986; Ingram and Reid 1987; Martin *et al.* 1999; Davidson *et al.* 2003) and 2 more involved in the response to the hormone (Peck *et al.* 2001). The closest significant SNP to *Le* in the present study (S5LG3_569851018) is 2 Mb away and it was associated with leaf area and internode 2–3. Eight other SNPs were associated with internode length, illustrating that more than 1 gene is responsible for the trait.

Using biparental populations, Smitchger and Weeden (2019) found that a QTL on chr2(LG1) associated with seed size, side branch diameter, leaf length, main stem diameter, compressed side branch thickness, and compressed main stem. A QTL for seed size in a similar position was previously reported and called Tsw1.1, so Smitchger and Weeden (2019) called the QTL Putative Tsw1.1. We identified a significant SNP (S2LG1_353493) for internode 2–3 at 1.09 Mb distance from Putative Tsw1.1.

Marker S5LG3_544595701, which here was significantly associated with leaf area and stem diameter, is 0.23 Mb from marker Chr5LG3_572669963 which was associated with reproductive stem length by Tafesse *et al.* (2020) in a GWAS that used a different population than the present study. Since these traits are both related to growth parameters and could be affected by similar processes, the markers could be associated with the same gene or a gene cluster in the region. We also found a marker (S5LG3_569851018) associated with leaf area to be 0.06 Mb from another marker (chr5LG3_569788697) previously associated with leaf chlorophyll concentration (Tafesse *et al.* 2020).

Ashtari Mahini *et al.* (2020) used a biparental population to evaluate resistance to *Sclerotinia sclerotium* in peas in controlled conditions and found the same QTL to be associated with internode length and 2 measures of disease resistance. The allele conferring resistance was also associated with shorter plant stature. We found that SNP S1LG6_369964198 on chr1 (LG6) had the minor allele associated with less disease (smaller sAUDPC), smaller leaf area, and thinner stem. This SNP in conjunction with the positive correlation of growth traits and sAUDPC (Fig. 1) is a strong indication that growth parameters and disease resistance can be affected by the same genes. This is not a new phenomenon, disease resistance genes might come at a metabolic cost for plants (Karasov *et al.* 2017), which could explains the results observed in this study that genotypes with smaller leaves have less disease.

## Data availability

GBS data aligned with the reference genome is available at https://github.com/selizpowers/GWAS (Powers *et al.* 2021). All phenotypic data are available in Supplementary Table 5.

Supplemental material is available at *G3* online.

## Supplementary Material

jkac168_Supplemental_Figure_S1

jkac168_Supplemental_Table_1

jkac168_Supplemental_Table_2

jkac168_Supplemental_Table_3

jkac168_Supplemental_Table_4

jkac168_Supplemental_Table_5

## References

[jkac168-B1] Achard P , Genschik P. Releasing the brakes of plant growth: how GAs shutdown DELLA proteins. J Exp Bot. 2009;60(4):1085–1092. 10.1093/JXB/ERN301. 19043067

[jkac168-B2] Ashtari Mahini R , Kumar Elias A, Elias M, Fiedler JD, Porter LD, McPhee KE. Analysis and Identification of QTL for Resistance to *Sclerotinia sclerotiorum* in pea (*Pisum sativum* L.). Front Genet. 2020;11:587968. 10.3389/fgene.2020.587968. 33329732 PMC7710873

[jkac168-B3] Bates D , Mächler M, Bolker B, Walker S. Fitting linear mixed-effects models using lme4. J Stat Softw. 2015;67(1):1–48. 10.18637/jss.v067.i01.

[jkac168-B4] Bradbury PJ , Zhang Z, Kroon DE, Casstevens TM, Ramdoss Y, Buckler ES. TASSEL: software for association mapping of complex traits in diverse samples. Bioinformatics. 2007;23(19):2633–2635. 10.1093/bioinformatics/btm308. 17586829

[jkac168-B5] Bretag TW , Keane PJ, Price TV. Effect of Ascochyta blight on the grain yield of field peas (*Pisum sativum* L.) grown in southern Australia. Aust J Exp Agric. 1995;35(4):531–536. 10.1071/EA9950531.

[jkac168-B6] Bretag TW , Keane PJ, Price TV. The epidemiology and control of Ascochyta blight in field peas: a review. Aust J Agric Res. 2006;57(8):883. 10.1071/AR05222.

[jkac168-B7] Brown JK , Rant JC. Fitness costs and trade-offs of disease resistance and their consequences for breeding arable crops. Plant Pathol. 2013;62(S1):83–95. 10.1111/ppa.12163.

[jkac168-B8] Butler DG , Cullis BR, Gilmour AR, Gogel BJ, Thompson R. 2018. “ASReml-R Reference Manual Version 4.” *ASReml-R Reference Manual*. Hemel Hempstead, UK: VSN International Ltd. https://asreml.kb.vsni.co.uk/knowledge-base/asreml_r_documentation/

[jkac168-B9] Campbell CL , Madden LV. Introduction to Plant Disease Epidemiology. New York: John Wiley & Sons; 1990. p. 192–194.

[jkac168-B10] Carrillo E , Rubiales D, Pérez-de-Luque A, Fondevilla S. Characterization of mechanisms of resistance against *Didymella pinodes* in Pisum spp. Eur J Plant Pathol. 2013;135(4):761–769. 10.1007/s10658-012&ndash;0116-0.

[jkac168-B11] Carrillo E , Satovic Z, Aubert G, Boucherot K, Rubiales D, Fondevilla S. Identification of quantitative trait loci and candidate genes for specific cellular resistance responses against *Didymella pinodes* in pea. Plant Cell Rep. 2014;33(7):1133–1145. 10.1007/s00299-014&ndash;1603-x. 24706065

[jkac168-B12] Castillejo M-Á , Fondevilla-Aparicio S, Fuentes-Almagro C, Rubiales D. Quantitative analysis of target peptides related to resistance against Ascochyta blight (*Peyronellaea pinodes*) in pea. J Proteome Res. 2020;19(3):1000–1012. 10.1021/acs.jproteome.9b00365. 32040328

[jkac168-B13] Davidson SE , Elliott RC, Helliwell CA, Poole AT, Reid JB. The pea gene NA encodes ent-kaurenoic acid oxidase. Plant Physiol. 2003;131(1):335–344. 10.1104/pp.012963. 12529541 PMC166813

[jkac168-B14] Ellis TH , Noel JM, Hofer GM, Timmerman-Vaughan CJ, Coyne RP Hellens. Mendel, 150 years on. Trends Plant Sci. 2011;16(11):590–596. 10.1016/j.tplants.2011.06.006. 21775188

[jkac168-B15] Fondevilla S , Almeida NF, Satovic Z, Rubiales D, Vaz Patto MC, Cubero JI, Torres AM. Identification of common genomic regions controlling resistance to *Mycosphaerella pinodes*, earliness and architectural traits in different pea genetic backgrounds. Euphytica. 2011;182(1):43–52. 10.1007/s10681-011&ndash;0460-8.

[jkac168-B16] Fondevilla S , Ávila CM, Cubero JI, Rubiales D. Response to *Mycosphaerella pinodes* in a germplasm collection of Pisum spp. Plant Breed. 2005;124(3):313–315. 10.1111/j.1439-0523.2005.01104.x.

[jkac168-B17] Fondevilla S , Fernández-Romero MD, Satovic Z, Rubiales D. Expressional and positional candidate genes for resistance to *Peyronellaea pinodes* in pea. Euphytica. 2018;214(12):1–14. 10.1007/s10681-018&ndash;2316-y.

[jkac168-B18] Fondevilla S , Küster H, Krajinski F, Cubero JI, Rubiales D. Identification of genes differentially expressed in a resistant reaction to *Mycosphaerella pinodes* in pea using microarray technology. BMC Genomics. 2011;12:28. 10.1186/1471&ndash;2164-12&ndash;28. 21226971 PMC3027157

[jkac168-B19] Fondevilla S , Satovic Z, Rubiales D, Moreno MT, Torres AM. Mapping of quantitative trait loci for resistance to *Mycosphaerella pinodes* in *Pisum sativum* subsp. Syriacum. Mol Breed. 2008;21(4):439–454. 10.1007/s11032-007&ndash;9144-4.

[jkac168-B20] Gali KK , Sackville A, Tafesse EG, Lachagari VBR, McPhee K, Hybl M, Mikić A, Smýkal P, McGee R, Burstin J, et al Genome-wide association mapping for agronomic and seed quality traits of field pea (*Pisum sativum* L.). Front Plant Sci. 2019;10:1538. 10.3389/fpls.2019.01538. 31850030 PMC6888555

[jkac168-B21] Holdsworth WL , Gazave E, Cheng P, Myers JR, Gore MA, Coyne CJ, McGee RJ, Mazourek M. A community resource for exploring and utilizing genetic diversity in the USDA pea single plant plus collection. Hortic Res. 2017;4(1):Article number: 17017. 10.1038/hortres.2017.17. PMC5405346 28503311

[jkac168-B22] Huang M , Liu X, Zhou Y, Summers RM, Zhang Z. BLINK: a package for the next level of genome-wide association studies with both individuals and markers in the millions. GigaScience. 2019;8(2):1–12. 10.1093/gigascience/giy154. PMC6365300 30535326

[jkac168-B23] Ingram TJ , Reid JB. Internode length in Pisum. Plant Physiol. 1987;83(4):1048–1053. https://doi-org.prox.lib.ncsu.edu/10.1111/j.1399–3054.1986.tb05945.x. 16665322 10.1104/pp.83.4.1048 PMC1056499

[jkac168-B24] Jha AB , Gali KK, Tar’an B, Warkentin TD. Fine mapping of QTLs for Ascochyta blight resistance in pea using heterogeneous inbred families. Front Plant Sci. 2017;8:1–12. 10.3389/fpls.2017.00765. 28536597 PMC5422545

[jkac168-B25] Jha AB , Warkentin TD, Gurusamy V, Tar'an B, Banniza S. Identification of Mycosphaerella blight resistance in wild Pisum species for use in pea breeding. Crop Sci. 2012;52(6):2462–2468. 10.2135/cropsci2012.04.0242.

[jkac168-B26] Karasov TL , Chae E, Herman JJ, Bergelson J. Mechanisms to mitigate the trade-off between growth and defense. Plant Cell. 2017;29(4):666–680. 10.1105/tpc.16.00931. 28320784 PMC5435432

[jkac168-B27] Khan TN , Timmerman-Vaughan GM, Rubiales D, Warkentin TD, Siddique KH, Erskine W, Barbetti MJ. *Didymella pinodes* and its management in field pea: challenges and opportunities. Field Crops Res. 2013;148:61–77. 10.1016/J.FCR.2013.04.003.

[jkac168-B28] Korte A , Farlow A. The advantages and limitations of trait analysis with GWAS: a review. Plant Methods. 2013;9(1):29. 10.1186/1746&ndash;4811-9&ndash;29. 23876160 PMC3750305

[jkac168-B29] Kreplak J , Madoui M-A, Cápal P, Novák P, Labadie K, Aubert G, Bayer PE, Gali KK, Syme RA, Main D, et al A Reference genome for pea provides insight into legume genome evolution. Nature Genet. 2019;51(9):1411–1422. 10.1038/s41588-019&ndash;0480-1. 31477930

[jkac168-B30] Lester DR , Ross JJ, Davies PJ, Reid J 6. Mendel’s stem length gene (Le) encodes a gibberellin 3p-hydroxylase. Plant Cell. 1997;9(8):1435–1443. https://academic.oup.com/plcell/article/9/8/1435/5986479. 9286112 10.1105/tpc.9.8.1435 PMC157009

[jkac168-B31] Lipka AE , Tian F, Wang Q, Peiffer J, Li M, Bradbury PJ, Gore MA, Buckler ES, Zhang Z. GAPIT: genome association and prediction integrated tool. Bioinformatics. 2012;28(18):2397–2399. 10.1093/BIOINFORMATICS/BTS444. 22796960

[jkac168-B32] Liu N , Xu S, Yao X, Zhang G, Mao W, Hu Q, Feng Z, Gong Y, Feng Z, Gong Y. Studies on the control of Ascochyta blight in field peas (*Pisum sativum* L.) caused by *Ascochyta pinodes* in Zhejiang Province, China. Front Microbiol. 2016;7:481. 10.3389/fmicb.2016.00481. 27148177 PMC4828446

[jkac168-B33] Martin DN , Proebsting WM, Hedden P. The SLENDER gene of pea encodes a gibberellin 2-oxidase. Plant Physiol. 1999;121(3):775–781. 10.1104/pp.121.3.775. 10557225 PMC59439

[jkac168-B34] Owati A , Agindotan B, Burrows M. Characterization of fungal species associated with Ascochyta blight of dry pea in Montana and North America and development of a differential medium for their detection. Plant Health Progress. 2020;21(4):262–271. 10.1094/PHP-05&ndash;20-0037-RS.

[jkac168-B35] Parihar AK , Dixit GP, Bohra A, Sen Gupta D, Singh AK, Kumar N, Singh D, Singh NP. Genetic advancement in dry pea (*Pisum sativum* L.): retrospect and prospect. In: Gosal SS, Wani SH (editors). Accelerated Plant Breeding, Volume 3. Cham: Springer. https://doi.org/10.1007/978-3-030-47306-8_10.

[jkac168-B36] Parr M , Grossman JM, Reberg-Horton SC, Brinton C, Crozier C. Nitrogen delivery from legume cover crops in no-till organic corn production. Agron J. 2011;103(6):1578–1590. 10.2134/agronj2011.0007.

[jkac168-B37] Peck DM , McDonald CK, Davidson JA. 2001. Blackspot survival in soil and stubble and aerial dissemination through the season. In: 10th Australian Agronomy Conference. Hobart, Tasmania.

[jkac168-B38] Powers S , Boatwright JL, Thavarajah D. Genome-wide association studies of mineral and phytic acid concentrations in pea (*Pisum sativum* L.) to evaluate biofortification potential. G3 (Bethesda). 2021;11(9):jkab227. https://doi.org/10.1093/g3journal/jkab227. 34544130 10.1093/g3journal/jkab227 PMC8496233

[jkac168-B39] Prioul S , Frankewitz A, Deniot G, Morin G, Baranger A. Mapping of quantitative trait loci for partial resistance to *Mycosphaerella pinodes* in pea (*Pisum sativum* L.), at the seedling and adult plant stages. Theor Appl Genet. 2004;108(7):1322–1334. 10.1007/s00122-003&ndash;1543-2. 14968300

[jkac168-B40] Prioul-Gervais S , Deniot G, Receveur EM, Frankewitz A, Fourmann M, Rameau C, Pilet-Nayel ML, Baranger A. Candidate genes for quantitative resistance to *Mycosphaerella pinodes* in pea (*Pisum sativum* L.). Theor Appl Genet. 2007;114(6):971–984. 10.1007/s00122-006&ndash;0492-y. 17265025

[jkac168-B41] Rasband WS. ImageJ. Bethesda (MD): U.S. National Institutes of Health; 1997. https://imagej.nih.gov/ij/.

[jkac168-B42] Rawal V , Navarro DK. The Global Economy of Pulses. Rome: FAO; 2019. 10.4060/i7108en.

[jkac168-B43] Reid JB , Potts WC. Internode length in Pisum. Two further mutants, Lh and Ls, with reduced gibberellin synthesis, and a gibberellin insensitive mutant, Lk. Physiol Plant. 1986;66(3):417–426. 10.1111/j.1399&ndash;3054.1986.tb05945.x.

[jkac168-B44] Rubiales D , Fondevilla S, Chen W, Gentzbittel L, Higgins TJ, Castillejo MA, Singh KB, Rispail N. Achievements and challenges in legume breeding for pest and disease resistance. Crit Revi Plant Sci. 2015;34(1–3):195–236. 10.1080/07352689.2014.898445.

[jkac168-B45] Rubiales D , González Bernal MJ, Warkentin T, Bueckert T, Vaz Patto MC, McPhee K, McGee R. Oxford University Press2019. “Advances in Pea Breeding.” In *Achieving Sustainable Cultivation of Vegetables*, edited by G. Hochmuth, pp. 2–31. Cambridge: Burleigh Dodds Science Publishing. 10.19103/AS.2019.0045.28.

[jkac168-B46] Schoeny A , Jumel S, Rouault F, Tivoli B. Assessment of Ascochyta blight (*Mycosphaerella pinodes*) on pea. *QuantiPest*. 1996. https://www6.inrae.fr/quantipest/Document-search-or-submission/List-of-documents/Assessment-of-ascochyta-blight-on-pea.

[jkac168-B47] Skoglund LG , Harveson RM, Chen W, Dugan F, Schwartz HF, Markell SG, Porter L, Burrows ML, Goswami R. Ascochyta blight of peas. Plant Health Progress. 2011;12(1). 10.1094/PHP-2011-0330-01-RS.

[jkac168-B48] Smitchger J , Weeden N. Quantitative trait loci controlling lodging resistance and other important agronomic traits in dry field peas. Crop Science. 2019;59(4):1442–1456. 10.2135/cropsci2018.04.0260.

[jkac168-B49] Tafesse EG , Gali KK, Lachagari VR, Bueckert R, Warkentin TD. Genome-wide association mapping for heat stress responsive traits in field pea. Int J Mol Sci. 2020;21(6):2043. 10.3390/ijms21062043. PMC7139655 32192061

[jkac168-B51] Timmerman-Vaughan GM , Frew TJ, Butler R, Murray S, Gilpin M, Falloon K, Johnston P, Lakeman MB, Russell A, Khan T. Validation of quantitative trait loci for Ascochyta blight resistance in pea (*Pisum sativum* L.), using populations from two crosses. Theor Appl Genet. 2004;109(8):1620–1631. 10.1007/s00122-004&ndash;1779-5. 15372153

[jkac168-B52] Timmerman-Vaughan GM , Frew TJ, Russell AC, Khan T, Butler R, Gilpin M, Murray S, Falloon K. QTL mapping of partial resistance to field epidemics of Ascochyta blight of pea. Crop Sci. 2002;42(6):2100–2111. 10.2135/cropsci2002.2100.

[jkac168-B53] Tivoli B , Baranger A, Muehlbauer FJ, Cooke BM. Ascochyta blights of grain legumes. Eur J Plant Pathol. 2007;119(1):59–76. https://doi.org/10.1007/978-1-4020–6065-6.

[jkac168-B54] Tivoli B , Béasse C, Lemarchand E, Masson E. Effect of Ascochyta blight (*Mycosphaerella pinodes*) on yield components of single pea (*Pisum sativum*) plants under field conditions. Ann Appl Biol. 1996;129(2):207–216. 10.1111/j.1744&ndash;7348.1996.tb05745.x.

[jkac168-B55] Wickham H. Ggplot2: Elegant Graphics for Data Analysis. New York (NY): Springer-Verlag; 2016. https://ggplot2.tidyverse.org.

[jkac168-B56] Wroth JM. Possible role for wild genotypes of Pisum spp. to enhance Ascochyta blight resistance in pea. Aust J Exp Agri. 1998;38(5):469–479. 10.1071/EA98024.

[jkac168-B57] Xue AG , Warkentin TD, Kenaschuk EO. Effects of timings of inoculation with *Mycosphaerella pinodes* on yield and seed infection of field pea. Can J Plant Sci. 1997;77(4):685–689. 10.4141/P96-150.

