# Supplementary material for: Genome-wide association study for morphological traits and resistance to Peryonella pinodes in the USDA pea single plant plus collection
Source: G3 (Bethesda). 2022 Jul 6;12(9):jkac168. doi: 10.1093/g3journal/jkac168 (PMC9434253; doi:10.1093/g3journal/jkac168)
Supplement: jkac168_Supplemental_Figure_S1 [file jkac168_supplemental_figure_s1.docx]

**Supplemental figure 1 –** Boxplot of distribution of disease ratings for each replicate. Different colors indicate different scoring date for each replicate


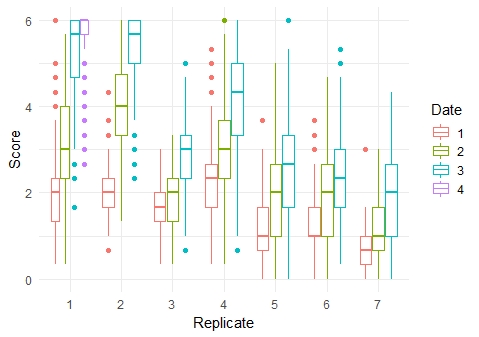


Box encompass the interquartile range, line in box shows the median, and whiskers expand to 1.5 times interquartile below and above quartile 1 and 3, respectively.
