# Supplementary material for: Genome-wide association study for morphological traits and resistance to Peryonella pinodes in the USDA pea single plant plus collection
Source: G3 (Bethesda). 2022 Jul 6;12(9):jkac168. doi: 10.1093/g3journal/jkac168 (PMC9434253; doi:10.1093/g3journal/jkac168)
Supplement: jkac168_Supplemental_Table_1 [file jkac168_supplemental_table_1.docx]

**Supplemental Table 1** - *Peryonella pinodes* isolate information

| **Isolate name** | **Host collected** | **Country of collection** | **Year of collection** |
| --- | --- | --- | --- |
| MP-1 | *Pisum sativum* | USA | 1995 |
| MP-3 | *Pisum sativum* | Morocco | 1989 |
| MP-5 | *Pisum sativum* | Canada | Unknown |
| MP-9 | *Pisum sativum* | New Zealand | 1996 |
| MP-25 | *Vicia faba* | Australia | Unknown |
| MP-29 | *Pisum sativum* | Bolivia | 1999 |
