# Supplementary material for: Genome-wide association study for morphological traits and resistance to Peryonella pinodes in the USDA pea single plant plus collection
Source: G3 (Bethesda). 2022 Jul 6;12(9):jkac168. doi: 10.1093/g3journal/jkac168 (PMC9434253; doi:10.1093/g3journal/jkac168)
Supplement: jkac168_Supplemental_Table_2 [file jkac168_supplemental_table_2.docx]

**Supplemental Table 2** - Correlation between replicates for 5 traits

| **Trait** |  | **Rep 2** | **Rep 3** | **Rep 4** | **Rep 5** | **Rep 6** | **Rep 7** |
| --- | --- | --- | --- | --- | --- | --- | --- |
| sAUDPC | **Rep 1** | 0.23 | 0.25 | 0.19 | 0.16 | 0.36 | 0.20 |
|  | **Rep 2** |  | 0.21 | 0.31 | 0.27 | 0.25 | 0.29 |
|  | **Rep 3** |  |  | 0.21 | 0.24 | 0.28 | 0.33 |
|  | **Rep 4** |  |  |  | 0.25 | 0.21 | 0.19 |
|  | **Rep 5** |  |  |  |  | 0.30 | 0.35 |
|  | **Rep 6** |  |  |  |  |  | 0.30 |
| Leaf area |  |  |  |  |  |  |  |
|  | **Rep 1** | 0.64 | 0.69 | 0.62 | 0.67 |  |  |
|  | **Rep 2** |  | 0.75 | 0.68 | 0.75 |  |  |
|  | **Rep 3** |  |  | 0.75 | 0.80 |  |  |
|  | **Rep 4** |  |  |  | 0.72 |  |  |
| Diameter |  |  |  |  |  |  |  |
|  | **Rep 1** | 0.77 | 0.59 | 0.73 | 0.74 |  |  |
|  | **Rep 2** |  | 0.7 | 0.77 | 0.76 |  |  |
|  | **Rep 3** |  |  | 0.58 | 0.68 |  |  |
|  | **Rep 4** |  |  |  | 0.71 |  |  |
| Internode 2-3 |  |  |  |  |  |  |  |
|  | **Rep 1** | 0.71 | 0.74 | 0.71 | 0.76 |  |  |
|  | **Rep 2** |  | 0.71 | 0.75 | 0.73 |  |  |
|  | **Rep 3** |  |  | 0.74 | 0.78 |  |  |
|  | **Rep 4** |  |  |  | 0.79 |  |  |
| Internode 5-6 |  |  |  |  |  |  |  |
|  | **Rep 1** | 0.74 | 0.74 | 0.76 | 0.71 |  |  |
|  | **Rep 2** |  | 0.8 | 0.83 | 0.75 |  |  |
|  | **Rep 3** |  |  | 0.84 | 0.82 |  |  |
|  | **Rep 4** |  |  |  | 0.81 |  |  |

All values are significant at P-value <0.001
