# Supplementary material for: Genome-wide association study for morphological traits and resistance to Peryonella pinodes in the USDA pea single plant plus collection
Source: G3 (Bethesda). 2022 Jul 6;12(9):jkac168. doi: 10.1093/g3journal/jkac168 (PMC9434253; doi:10.1093/g3journal/jkac168)
Supplement: jkac168_Supplemental_Table_3 [file jkac168_supplemental_table_3.docx]

**Supplemental Table 3** - Table of variances for all random model used to calculate BLUPs for each trait

| **Trait** | **Factor** | **Variance** |
| --- | --- | --- |
| **AUDPC** | rep | 0.126 |
|  | side:rep | 0.001 |
|  | number.nodes:rep | 0.006 |
|  | entry | 0.021 |
|  | tray:cart:rep | 0.009 |
|  | entry:rep | 0.015 |
|  | residual | 0.034 |
| **Leaf area** | entry:rep | 161294.5 |
|  | entry | 480920.3 |
|  | rep | 81408.3 |
|  | Residual | 41497.5 |
| **Diameter** | entry | 0.204 |
|  | rep | 0.001 |
|  | Residual | 0.080 |
| **Internode2-3** | entry:rep | 51.6 |
|  | entry | 129.8 |
|  | rep | 79.4 |
|  | Residual | 3.6 |
| **Internode5-6** | entry:rep | 59.9 |
|  | entry | 225.5 |
|  | rep | 81.2 |
|  | Residual | 3.5 |

Replicate(rep), side of growth chamber that the nested in replicate (side:rep), number of nodes in the plant at final score nested in replicate (number.nodes:rep), genotype (entry), interaction of genotype and replicate (entry:rep), and residual
