# Supplementary material for: Genome-wide association study for morphological traits and resistance to Peryonella pinodes in the USDA pea single plant plus collection
Source: G3 (Bethesda). 2022 Jul 6;12(9):jkac168. doi: 10.1093/g3journal/jkac168 (PMC9434253; doi:10.1093/g3journal/jkac168)
Supplement: jkac168_Supplemental_Table_4 [file jkac168_supplemental_table_4.docx]

**Supplemental Table 4** - Candidate genes found in 500Kb flanking of significant SNPs

| **SNP** | **Chr** | **SNP_Position** | **Traits** | **Gene** | **Gene_info** |
| --- | --- | --- | --- | --- | --- |
| S1LG6_26051507 | 1LG6 | 26051507 | sAUDPC | Psat1g018000 | Unknown |
| - | - | - | - | Psat1g017840 | Unknown |
| - | - | - | - | Psat1g017920 | Ubiquitin family |
| - | - | - | - | Psat1g018040 | S-adenosylmethionine synthetase + C-terminal domain |
| - | - | - | - | Psat1g018120 | SBP domain |
| - | - | - | - | Psat1g018080 | Late nodulin protein |
| - | - | - | - | Psat1g018160 | AWPM-19-like family |
| - | - | - | - | Psat1g018200 | Unknown |
| - | - | - | - | Psat1g018240 | Lysine methyltransferase |
| - | - | - | - | Psat1g018280 | Zinc-binding dehydrogenase |
| - | - | - | - | Psat1g018320 | Unknown |
| - | - | - | - | Psat1g018360 | F-box domain |
| - | - | - | - | Psat1g018400 | PPR repeat family |
| - | - | - | - | Psat1g018440 | Unknown |
| - | - | - | - | Psat1g018480 | Cell part |
| - | - | - | - | Psat1g018520 | F-box domain |
| - | - | - | - | Psat1g018600 | Unknown |
| - | - | - | - | Psat1g018560 | Unknown |
| - | - | - | - | Psat1g018640 | Cellular nucleobase + nucleoside + nucleotide and nucleic acid metabolic process |
| - | - | - | - | Psat1g018680 | JmjC domain + hydroxylase |
| - | - | - | - | Psat1g018720 | FAS1/BIgH3 domain profile. |
| - | - | - | - | Psat1g018760 | Soluble NSF attachment protein + SNAP |
| - | - | - | - | Psat1g018800 | GDSL-like Lipase/Acylhydrolase |
| S1LG6_31928165 | 1LG6 | 26051507 | Leaf area | Psat1g022160 | Unknown |
|  |  |  |  | Psat1g022200 | Glycerophosphoryl diester phosphodiesterase family |
|  |  |  |  | Psat1g022240 | Lipocalin-like domain |
|  |  |  |  | Psat1g022280 | Protein of unknown function (DUF1068) |
|  |  |  |  | Psat1g022320 | Response regulator receiver domain |
|  |  |  |  | Psat1g022360 | Calmodulin binding protein-like |
|  |  |  |  | Psat1g022400 | Unknown |
|  |  |  |  | Psat1g022440 | Serine carboxypeptidase |
|  |  |  |  | Psat1g022480 | YbaB/EbfC DNA-binding family |
|  |  |  |  | Psat1g022520 | Acyltransferase |
|  |  |  |  | Psat1g022560 | Oligosaccharyl transferase STT3 subunit |
|  |  |  |  | Psat1g022600 | Leucine rich repeat N-terminal domain |
|  |  |  |  | Psat1g022640 | Unknown |
|  |  |  |  | Psat1g022680 | Sugar (and other) transporter |
|  |  |  |  | Psat1g022720 | Von Willebrand factor + type A |
|  |  |  |  | Psat1g022760 | Transferase activity + transferring phosphorus-containing groups |
|  |  |  |  | Psat1g022840 | Adenylate kinase |
|  |  |  |  | Psat1g022880 | Unknown |
|  |  |  |  | Psat1g022920 | FAD dependent oxidoreductase |
|  |  |  |  | Psat1g022960 | E1-E2 ATPase |
|  |  |  |  | Psat1g023000 | Unknown |
|  |  |  |  | Psat1g023040 | Major Facilitator Superfamily |
|  |  |  |  | Psat1g023080 | Unknown |
|  |  |  |  | Psat1g023160 | Unknown |
| S1LG6_261934321 | 1LG6 | 261934321 | Leaf area, Stem diameter | Psat1g131720 | Protein kinase domain |
| - | - | - | - | Psat1g131400 | Nucleotide hydrolase |
| - | - | - | - | Psat1g131440 | Unknown |
| - | - | - | - | Psat1g131480 | Protein kinase domain |
| - | - | - | - | Psat1g131520 | Protein phosphatase 2C |
| - | - | - | - | Psat1g131560 | Unknown |
| - | - | - | - | Psat1g131600 | Unknown |
| - | - | - | - | Psat1g131640 | N-terminal domain of CBF1 interacting co-repressor CIR |
| - | - | - | - | Psat1g131680 | Regulation of biological process |
| - | - | - | - | Psat1g131760 | Unknown |
| - | - | - | - | Psat1g131800 | DDE superfamily endonuclease |
| - | - | - | - | Psat1g131840 | Sodium/hydrogen exchanger family |
| - | - | - | - | Psat1g131880 | Unknown |
| - | - | - | - | Psat1g131920 | Unknown |
| - | - | - | - | Psat1g131960 | Unknown |
| - | - | - | - | Psat1g132000 | Alpha mannosidase middle domain |
| - | - | - | - | Psat1g132040 | IQ motif profile |
| S1LG6_351220787 | 1LG6 | 351220787 | Internode 2-3 | Psat1g200280 | NUDIX domain |
| - | - | - | - | Psat1g200240 | NUDIX domain |
| - | - | - | - | Psat1g200320 | Magnesium chelatase + subunit ChlI |
| - | - | - | - | Psat1g200360 | IQ calmodulin-binding motif |
| - | - | - | - | Psat1g200400 | Ribosomal Proteins L2 + C-terminal domain |
| - | - | - | - | Psat1g200480 | Unknown |
| - | - | - | - | Psat1g200440 | Unknown |
| - | - | - | - | Psat1g200520 | Unknown |
| - | - | - | - | Psat1g200560 | Unknown |
| - | - | - | - | Psat1g200600 | Unknown |
| - | - | - | - | Psat1g200680 | Receptor family ligand binding region |
| - | - | - | - | Psat1g200640 | Oligonucleotide/oligosaccharide-binding (OB)-fold |
| - | - | - | - | Psat1g200720 | Receptor family ligand binding region |
| - | - | - | - | Psat1g200760 | Zinc knuckle |
| - | - | - | - | Psat1g200800 | GDSL-like Lipase/Acylhydrolase |
| - | - | - | - | Psat1g200840 | Unknown |
| - | - | - | - | Psat1g200880 | Unknown |
| - | - | - | - | Psat1g200920 | AP2 domain |
| - | - | - | - | Psat1g201000 | AP2 domain |
| - | - | - | - | Psat1g201040 | Unknown |
| - | - | - | - | Psat1g201080 | 1-(5-phosphoribosyl)-5-[(5-phosphoribosylamino)methylideneamino]imidazole-4-carboxamide isomerase activity |
| - | - | - | - | Psat1g201160 | Homeobox domain |
| - | - | - | - | Psat1g201200 | Unknown |
| - | - | - | - | Psat1g201360 | Glutaredoxin |
| - | - | - | - | Psat1g201240 | Ras family |
| - | - | - | - | Psat1g201400 | Unknown |
| - | - | - | - | Psat1g201280 | Unknown |
| - | - | - | - | Psat1g201320 | Protein kinase domain |
| - | - | - | - | Psat1g201440 | Casein kinase substrate phosphoprotein PP28 |
| S1LG6_369964198 | 1LG6 | 369964198 | Leaf area, sAUDPC, Stem diameter | Psat1g221440 | Unknown |
| - | - | - | - | Psat1g221480 | Arginine and glutamate-rich 1 |
| - | - | - | - | Psat1g221600 | Unknown |
| - | - | - | - | Psat1g221560 | Peroxidase |
| - | - | - | - | Psat1g221680 | Pentatricopeptide repeat |
| - | - | - | - | Psat1g221720 | START domain |
| - | - | - | - | Psat1g221760 | Magnesium transporter NIPA |
| - | - | - | - | Psat1g221800 | Intracellular non-membrane-bounded organelle |
| - | - | - | - | Psat1g221840 | Etoposide-induced protein 2.4 (EI24) |
| - | - | - | - | Psat1g221880 | TCP family transcription factor |
| - | - | - | - | Psat1g221920 | Zinc finger C2H2 type domain profile |
| - | - | - | - | Psat1g221960 | Ion transport protein |
| - | - | - | - | Psat1g222000 | Protein of unknown function (DUF793) |
| - | - | - | - | Psat1g222040 | Serine hydroxymethyltransferase |
| - | - | - | - | Psat1g222080 | T-complex protein 11 |
| - | - | - | - | Psat1g222160 | Phosphomethylpyrimidine kinase |
| - | - | - | - | Psat1g222200 | Protein kinase domain |
| - | - | - | - | Psat1g222120 | RQC domain |
| - | - | - | - | Psat1g222240 | 2OG-Fe(II) oxygenase superfamily |
| - | - | - | - | Psat1g222280 | Castor and Pollux + part of voltage-gated ion channel |
| - | - | - | - | Psat1g222320 | Unknown |
| - | - | - | - | Psat1g222360 | YT521-B-like domain |
| - | - | - | - | Psat1g222400 | Bromodomain |
| - | - | - | - | Psat1g222440 | Protein of unknown function (DUF679) |
| - | - | - | - | Psat1g222480 | Protein tyrosine kinase |
| - | - | - | - | Psat1g222520 | Unknown |
| - | - | - | - | Psat1g222560 | MAC/Perforin domain |
| - | - | - | - | Psat1g222600 | Unknown |
| S2LG1_353493 | 2LG1 | 353493 | Internode 2-3 | Psat2g000160 | Unknown |
| - | - | - | - | Psat2g000200 | DYW family of nucleic acid deaminases |
| - | - | - | - | Psat2g000240 | Histone deacetylase domain |
| - | - | - | - | Psat2g000280 | Queuine tRNA-ribosyltransferase |
| - | - | - | - | Psat2g000320 | SWIM zinc finger |
| - | - | - | - | Psat2g000360 | Unknown |
| - | - | - | - | Psat2g000400 | Sec7 domain |
| - | - | - | - | Psat2g000480 | Voltage gated chloride channel |
| - | - | - | - | Psat2g000440 | FAR1 DNA-binding domain |
| - | - | - | - | Psat2g000520 | Nucleotide-sugar transporter |
| - | - | - | - | Psat2g000560 | Unknown |
| - | - | - | - | Psat2g000600 | Protein kinase domain |
| - | - | - | - | Psat2g000640 | Mitochondrial carrier protein |
| - | - | - | - | Psat2g000680 | Homoserine kinase signature |
| - | - | - | - | Psat2g000800 | Homoserine kinase signature |
| - | - | - | - | Psat2g000840 | Cytoskeletal-regulatory complex EF hand |
| - | - | - | - | Psat2g000880 | Signal recognition particle 14kD protein |
| - | - | - | - | Psat2g000960 | Protein tyrosine kinase |
| - | - | - | - | Psat2g001000 | Protein kinase domain |
| - | - | - | - | Psat2g001080 | Niemann-Pick C1 N terminus |
| - | - | - | - | Psat2g001120 | RWP-RK domain |
| S2LG1_528924 | 2LG1 | 528924 | Internode 2-3 | Psat2g000160 | Unknown |
| - | - | - | - | Psat2g000200 | DYW family of nucleic acid deaminases |
| - | - | - | - | Psat2g000240 | Histone deacetylase domain |
| - | - | - | - | Psat2g000280 | Queuine tRNA-ribosyltransferase |
| - | - | - | - | Psat2g000320 | SWIM zinc finger |
| - | - | - | - | Psat2g000360 | Unknown |
| - | - | - | - | Psat2g000400 | Sec7 domain |
| - | - | - | - | Psat2g000480 | Voltage gated chloride channel |
| - | - | - | - | Psat2g000440 | FAR1 DNA-binding domain |
| - | - | - | - | Psat2g000520 | Nucleotide-sugar transporter |
| - | - | - | - | Psat2g000560 | Unknown |
| - | - | - | - | Psat2g000600 | Protein kinase domain |
| - | - | - | - | Psat2g000640 | Mitochondrial carrier protein |
| - | - | - | - | Psat2g000680 | Homoserine kinase signature |
| - | - | - | - | Psat2g000800 | Homoserine kinase signature |
| - | - | - | - | Psat2g000840 | Cytoskeletal-regulatory complex EF hand |
| - | - | - | - | Psat2g000880 | Signal recognition particle 14kD protein |
| - | - | - | - | Psat2g000960 | Protein tyrosine kinase |
| - | - | - | - | Psat2g001000 | Protein kinase domain |
| - | - | - | - | Psat2g001080 | Niemann-Pick C1 N terminus |
| - | - | - | - | Psat2g001120 | RWP-RK domain |
| - | - | - | - | Psat2g001160 | Transferase activity + transferring phosphorus-containing groups |
| - | - | - | - | Psat2g001200 | Unknown |
| - | - | - | - | Psat2g001240 | C2H2-type zinc finger |
| - | - | - | - | Psat2g001280 | Histidine phosphatase superfamily (branch 1) |
| - | - | - | - | Psat2g001320 | MatE |
| - | - | - | - | Psat2g001360 | GDP-fucose protein O-fucosyltransferase |
| S2LG1_3521266 | 2LG1 | 3521266 | Internode 2-3 | Psat2g004200 | Unknown |
| - | - | - | - | Psat2g004240 | Unknown |
| - | - | - | - | Psat2g004280 | Unknown |
| - | - | - | - | Psat2g004320 | Unknown |
| - | - | - | - | Psat2g004360 | CHASE domain |
| - | - | - | - | Psat2g004400 | Arabidopsis thaliana 130.7kDa hypothetical protein signature |
| - | - | - | - | Psat2g004440 | FAS1 domain |
| - | - | - | - | Psat2g004480 | Hsp90 protein |
| - | - | - | - | Psat2g004520 | Unknown |
| - | - | - | - | Psat2g004560 | Chlorophyll A-B binding protein |
| - | - | - | - | Psat2g004600 | SAM domain (Sterile alpha motif) |
| - | - | - | - | Psat2g004640 | Transcription factor + FAR1-related |
| - | - | - | - | Psat2g004680 | Ribosomal protein S26e |
| - | - | - | - | Psat2g004720 | Serine carboxypeptidase |
| - | - | - | - | Psat2g004760 | Domain of unknown function (DUF3444) |
| S2LG1_4685463 | 2LG1 | 4685463 | Leaf area | Psat2g004960 | E1-E2 ATPase |
| - | - | - | - | Psat2g005000 | Nup133 N terminal like |
| - | - | - | - | Psat2g005040 | WD40/YVTN repeat-like-containing domain |
| - | - | - | - | Psat2g005120 | Unknown |
| - | - | - | - | Psat2g005160 | Nucleotidyl transferase |
| - | - | - | - | Psat2g005200 | Plant phosphoribosyltransferase C-terminal |
| - | - | - | - | Psat2g005240 | Enolase + C-terminal TIM barrel domain |
| - | - | - | - | Psat2g005280 | F-box associated |
| - | - | - | - | Psat2g005320 | Neutral/alkaline non-lysosomal ceramidase + N-terminal |
| - | - | - | - | Psat2g005360 | Armadillo/beta-catenin-like repeat |
| - | - | - | - | Psat2g005480 | Transmembrane amino acid transporter protein |
| - | - | - | - | Psat2g005520 | Transmembrane amino acid transporter protein |
| - | - | - | - | Psat2g005600 | Raffinose synthase or seed imbibition protein Sip1 |
| - | - | - | - | Psat2g005560 | Cytochrome oxidase assembly protein |
| - | - | - | - | Psat2g005680 | Subtilase family |
| - | - | - | - | Psat2g005720 | Unknown |
| - | - | - | - | Psat2g005760 | Late embryogenesis abundant protein |
| - | - | - | - | Psat2g005800 | Fructose-bisphosphate aldolase class-I |
| - | - | - | - | Psat2g005840 | Nucleolar protein +Nop52 |
| - | - | - | - | Psat2g005880 | Nucleolar protein +Nop52 |
| - | - | - | - | Psat2g005920 | Uracil phosphoribosyltransferase |
| - | - | - | - | Psat2g005960 | Kinesin motor domain |
| S2LG1_367524526 | 2LG1 | 367524526 | Leaf area | Psat2g141560 | Unknown |
| - | - | - | - | Psat2g141600 | SET domain |
| - | - | - | - | Psat2g141640 | SET domain |
| - | - | - | - | Psat2g141680 | Protein of unknown function (DUF3464) |
| - | - | - | - | Psat2g141760 | Transcription factor regulating root and shoot growth via Pin3 |
| - | - | - | - | Psat2g141720 | Unknown |
| - | - | - | - | Psat2g141880 | Protein kinase domain |
| - | - | - | - | Psat2g141840 | Unknown |
| - | - | - | - | Psat2g141920 | Unknown |
| - | - | - | - | Psat2g142000 | Putative S-adenosyl-L-methionine-dependent methyltransferase |
| - | - | - | - | Psat2g142040 | Unknown |
| - | - | - | - | Psat2g142080 | Replication factor-A C terminal domain |
| S4LG4_14403384 | 4LG4 | 14403384 | Leaf area | Psat4g010360 | Unknown |
|  |  |  |  | Psat4g010400 | Alg9-like mannosyltransferase family |
|  |  |  |  | Psat4g010440 | Transferase activity + transferring glycosyl groups |
|  |  |  |  | Psat4g010480 | Acetyltransferase (GNAT) family |
|  |  |  |  | Psat4g010520 | Ankyrin repeats (3 copies) |
|  |  |  |  | Psat4g010560 | Unknown |
|  |  |  |  | Psat4g010600 | Sugar (and other) transporter |
|  |  |  |  | Psat4g010640 | Ring finger domain |
| S4LG4_416335752 | 4LG4 | 416335752 | Internode 5-6 | Psat4g203760 | Unknown |
| - | - | - | - | Psat4g203800 | AP2 domain |
| - | - | - | - | Psat4g203840 | TPL-binding domain in jasmonate signalling |
| - | - | - | - | Psat4g203880 | Unknown |
| - | - | - | - | Psat4g203920 | ER lumen protein retaining receptor |
| - | - | - | - | Psat4g203960 | ER lumen protein retaining receptor |
| - | - | - | - | Psat4g204000 | ER lumen protein retaining receptor |
| - | - | - | - | Psat4g204040 | Cytochrome P450 |
| - | - | - | - | Psat4g204080 | Unknown |
| - | - | - | - | Psat4g204120 | Cytochrome P450 |
| - | - | - | - | Psat4g204160 | Unknown |
| - | - | - | - | Psat4g204200 | Leucine rich repeat |
| - | - | - | - | Psat4g204240 | Domain of unknown function (DUF3444) |
| - | - | - | - | Psat4g204400 | 1-deoxy-D-xylulose 5-phosphate reductoisomerase |
| - | - | - | - | Psat4g204280 | Unknown |
| - | - | - | - | Psat4g204320 | Transferase activity + transferring phosphorus-containing groups |
| - | - | - | - | Psat4g204360 | GTP cyclohydrolase I |
| - | - | - | - | Psat4g204440 | Vacuolar sorting protein 39 domain 2 |
| - | - | - | - | Psat4g204480 | Hydrolase activity + hydrolyzing O-glycosyl compounds |
| - | - | - | - | Psat4g204520 | Protein kinase domain |
| - | - | - | - | Psat4g204600 | Protein of unknown function (DUF760) |
| - | - | - | - | Psat4g204640 | Hydrolase activity + hydrolyzing O-glycosyl compounds |
| - | - | - | - | Psat4g204680 | Glycosyl hydrolases family 17 |
| - | - | - | - | Psat4g204720 | E1-E2 ATPase |
| - | - | - | - | Psat4g204760 | FKBP-type peptidyl-prolyl cis-trans isomerase |
| - | - | - | - | Psat4g204800 | FKBP-type peptidyl-prolyl cis-trans isomerase |
| - | - | - | - | Psat4g204920 | FKBP-type peptidyl-prolyl cis-trans isomerase |
| - | - | - | - | Psat4g204880 | Unknown |
| - | - | - | - | Psat4g204960 | Ribosomal protein S8e |
| - | - | - | - | Psat4g205000 | Glycosyltransferase like family 2 |
| S5LG3_198269966 | 5LG3 | 198269966 | sAUDPC | Psat5g110120 | Domain of unknown function (DUF4033) |
| - | - | - | - | Psat5g110160 | Lung seven transmembrane receptor |
| - | - | - | - | Psat5g110200 | Glycosyl hydrolase family 9 |
| - | - | - | - | Psat5g110240 | Glycosyl hydrolase family 9 |
| - | - | - | - | Psat5g110280 | Intron-binding protein aquarius N-terminus |
| - | - | - | - | Psat5g110320 | Ribosomal protein L11 + N-terminal domain |
| - | - | - | - | Psat5g110360 | Retroviral aspartyl protease |
| - | - | - | - | Psat5g110400 | Tim10/DDP family zinc finger |
| - | - | - | - | Psat5g110440 | Subtilase family |
| - | - | - | - | Psat5g110480 | Ring finger domain |
| - | - | - | - | Psat5g110520 | Subtilase family |
| - | - | - | - | Psat5g110640 | Sodium/calcium exchanger protein |
| - | - | - | - | Psat5g110680 | Myb-like DNA-binding domain |
| - | - | - | - | Psat5g110720 | Nucleotidyl transferase |
| - | - | - | - | Psat5g110760 | Unknown |
| - | - | - | - | Psat5g110600 | YL1 nuclear protein C-terminal domain |
| - | - | - | - | Psat5g110800 | PPR repeat family |
| - | - | - | - | Psat5g110840 | FAM91 N-terminus |
| - | - | - | - | Psat5g110880 | Unknown |
| - | - | - | - | Psat5g110920 | TFIIS helical bundle-like domain |
| S5LG3_544595701 | 5LG3 | 544595701 | Leaf area, Stem diameter | Psat5g279720 | Superfamilies 1 and 2 helicase C-terminal domain profile. |
| - | - | - | - | Psat5g279520 | PIGA (GPI anchor biosynthesis) |
| - | - | - | - | Psat5g279560 | Protein kinase domain |
| - | - | - | - | Psat5g279600 | Barwin-related endoglucanase |
| - | - | - | - | Psat5g279800 | Unknown |
| - | - | - | - | Psat5g279840 | Unknown |
| - | - | - | - | Psat5g279880 | Unknown |
| - | - | - | - | Psat5g279920 | Unknown |
| - | - | - | - | Psat5g279960 | Unknown |
| S5LG3_555981910 | 5LG3 | 555981910 | Leaf area | Psat5g289200 | Protein tyrosine kinase |
| - | - | - | - | Psat5g289240 | FAR1 DNA-binding domain |
| - | - | - | - | Psat5g289280 | Ubiquitin signature |
| - | - | - | - | Psat5g289320 | Ubiquitin signature |
| - | - | - | - | Psat5g289400 | Domain of unknown function (DUF4504) |
| - | - | - | - | Psat5g289520 | B-box zinc finger |
| - | - | - | - | Psat5g289600 | Strictosidine synthase |
| - | - | - | - | Psat5g289360 | PfkB family carbohydrate kinase |
| - | - | - | - | Psat5g289440 | Domain of unknown function (DUF4504) |
| - | - | - | - | Psat5g289480 | Unknown gene |
| - | - | - | - | Psat5g289560 | Leucine rich repeat |
| - | - | - | - | Psat5g289640 | Electron transfer flavoprotein-ubiquinone oxidoreductase + 4Fe-4S |
| - | - | - | - | Psat5g289680 | Regulation of cellular nucleobase |
| - | - | - | - | Psat5g289720 | Hydroxyacylglutathione hydrolase C-terminus |
| - | - | - | - | Psat5g289760 | BZIP transcription factor |
| - | - | - | - | Psat5g289880 | Lipoxygenase |
| - | - | - | - | Psat5g289920 | Linker histone H1 and H5 family |
| - | - | - | - | Psat5g289960 | Metal-dependent phosphohydrolase + HD subdomain |
| - | - | - | - | Psat5g289800 | Lipoxygenase |
| - | - | - | - | Psat5g289840 | Formin Homology 2 Domain |
| - | - | - | - | Psat5g290000 | Unknown gene |
| - | - | - | - | Psat5g290040 | Unknown gene |
| - | - | - | - | Psat5g290080 | Unknown gene |
| - | - | - | - | Psat5g290120 | Late embryogenesis abundant protein |
| - | - | - | - | Psat5g290200 | Unknown gene |
| - | - | - | - | Psat5g290160 | Chromo (CHRromatin Organisation MOdifier) domain |
| - | - | - | - | Psat5g290280 | Protein kinase domain |
| S5LG3_561689517 | 5LG3 | 561689517 | Internode 2-3 | Psat5g294800 | Peptidase family M20/M25/M40 |
| - | - | - | - | Psat5g294480 | Unknown |
| - | - | - | - | Psat5g294520 | Unknown |
| - | - | - | - | Psat5g294560 | Hydrolase activity + acting on acid anhydrides + catalyzing transmembrane movement of substances |
| - | - | - | - | Psat5g294600 | Helix-loop-helix DNA-binding domain |
| - | - | - | - | Psat5g294680 | TRAM domain profile. |
| - | - | - | - | Psat5g294640 | TRNA (Uracil-5-)-methyltransferase |
| - | - | - | - | Psat5g294720 | Carbohydrate-binding protein of the ER |
| - | - | - | - | Psat5g294760 | WD40/YVTN repeat-like-containing domain |
| - | - | - | - | Psat5g294840 | Unknown |
| - | - | - | - | Psat5g294880 | Peptidase family M20/M25/M40 |
| - | - | - | - | Psat5g294920 | Peptidase family M20/M25/M40 |
| - | - | - | - | Psat5g294960 | Peptidase family M20/M25/M40 |
| - | - | - | - | Psat5g295040 | GDSL-like Lipase/Acylhydrolase |
| - | - | - | - | Psat5g295000 | Mannose-binding lectin |
| - | - | - | - | Psat5g295080 | Unknown |
| - | - | - | - | Psat5g295160 | Unknown |
| S5LG3_569851018 | 5LG3 | 569851018 | Internode 2-3, Leaf area | Psat5g301000 | Asp/Glu/Hydantoin racemase |
| - | - | - | - | Psat5g301040 | Zinc finger C-x8-C-x5-C-x3-H type (and similar) |
| - | - | - | - | Psat5g300960 | Embryo-specific protein 3 + (ATS3) |
| - | - | - | - | Psat5g301120 | Protein of unknown function + DUF538 |
| - | - | - | - | Psat5g301160 | Cotton fibre expressed protein |
| - | - | - | - | Psat5g301200 | Polypeptide deformylase |
| - | - | - | - | Psat5g301240 | Ribosomal protein S28e |
| - | - | - | - | Psat5g301320 | Unknown |
| - | - | - | - | Psat5g301360 | Domain of unknown function (DUF4666) |
| - | - | - | - | Psat5g301280 | Glycosyl hydrolases family 28 |
| - | - | - | - | Psat5g301400 | Nup93/Nic96 |
| - | - | - | - | Psat5g301440 | Embryo-specific protein 3 + (ATS3) |
| - | - | - | - | Psat5g301480 | Unknown |
| - | - | - | - | Psat5g301520 | Glyoxalase/bleomycin resistance protein/dioxygenase |
| - | - | - | - | Psat5g301560 | Alba |
| - | - | - | - | Psat5g301600 | PPR repeat family |
| - | - | - | - | Psat5g301640 | PPR repeat family |
| - | - | - | - | Psat5g301680 | Unknown |
| - | - | - | - | Psat5g301720 | Peptidase S24-like |
| - | - | - | - | Psat5g301760 | Protein of unknown function + DUF393 |
| - | - | - | - | Psat5g301800 | Rhomboid family |
| - | - | - | - | Psat5g301840 | Glycosyl hydrolases family 28 |
| - | - | - | - | Psat5g301880 | Embryo-specific protein 3 + (ATS3) |
| - | - | - | - | Psat5g301920 | Establishment of localization |
| - | - | - | - | Psat5g301960 | Cellular component organization |
| - | - | - | - | Psat5g302000 | LURP-one-related |
| - | - | - | - | Psat5g302040 | Phospholipase D C terminal |
| - | - | - | - | Psat5g302080 | Nucleic acid-binding + OB-fold-like |
| - | - | - | - | Psat5g302120 | Unknown |
| S5LG3_572900348 | 5LG3 | 572900348 | Internode 2-3, Internode 5-6 | Psat5g303240 | PX domain |
| - | - | - | - | Psat5g303280 | Regulation of biological process |
| - | - | - | - | Psat5g303320 | Major Facilitator Superfamily |
| - | - | - | - | Psat5g303480 | Intracellular membrane-bounded organelle |
| - | - | - | - | Psat5g303560 | CUE domain |
| - | - | - | - | Psat5g303640 | Glyoxalase/Bleomycin resistance protein/Dioxygenase superfamily |
| - | - | - | - | Psat5g303680 | Sterile alpha motif homology |
| - | - | - | - | Psat5g303720 | Transmembrane amino acid transporter protein |
| - | - | - | - | Psat5g303760 | Cellular biosynthetic process |
| - | - | - | - | Psat5g303800 | Nodulin-like |
| - | - | - | - | Psat5g303840 | Gamma-glutamyl cyclotransferase + AIG2-like |
| - | - | - | - | Psat5g303880 | Sterile alpha motif homology |
| - | - | - | - | Psat5g303920 | Unknown |
| - | - | - | - | Psat5g303960 | Leucine Rich repeat |
| - | - | - | - | Psat5g304000 | BRCA1-associated protein 2 |
| - | - | - | - | Psat5g304040 | Isocitrate/isopropylmalate dehydrogenase |
| - | - | - | - | Psat5g304080 | TCP-1/cpn60 chaperonin family |
| - | - | - | - | Psat5g304160 | KIX domain |
| - | - | - | - | Psat5g304120 | PPR repeat family |
| - | - | - | - | Psat5g304200 | KIX domain |
| - | - | - | - | Psat5g304240 | Proline rich extensin signature |
| - | - | - | - | Psat5g304280 | Unknown |
| - | - | - | - | Psat5g304320 | Unknown |
| - | - | - | - | Psat5g304360 | Ribosomal protein S7p/S5e |
| S7LG7_37540311 | 7LG7 | 37540311 | sAUDPC | Psat7g024040 | Unknown |
| - | - | - | - | Psat7g024080 | Reverse transcriptase-like |
| - | - | - | - | Psat7g024120 | Reverse transcriptase-like |
| - | - | - | - | Psat7g024160 | SnoaL-like domain |
| - | - | - | - | Psat7g024200 | Unknown |
| - | - | - | - | Psat7g024240 | Acyltransferase C-terminus |
| - | - | - | - | Psat7g024280 | ATPase family associated with various cellular activities (AAA) |
| - | - | - | - | Psat7g024320 | Recoverin family signature |
| - | - | - | - | Psat7g024360 | Recoverin family signature |
| - | - | - | - | Psat7g024400 | Phosphatidylinositol 3- and 4-kinase |
| - | - | - | - | Psat7g024440 | Late embryogenesis abundant protein |
| - | - | - | - | Psat7g024480 | Unknown |
| - | - | - | - | Psat7g024560 | Plant transposase (Ptta/En/Spm family) |
| - | - | - | - | Psat7g024600 | Wall-associated receptor kinase galacturonan-binding |
| - | - | - | - | Psat7g024640 | Ring finger domain |
| - | - | - | - | Psat7g024680 | Ring finger domain |
| - | - | - | - | Psat7g024720 | Development and cell death domain |
| - | - | - | - | Psat7g024760 | Unknown |
| - | - | - | - | Psat7g024800 | Glutaredoxin |
| S7LG7_336950420 | 7LG7 | 336950420 | sAUDPC | Psat7g179160 | Unknown |
| - | - | - | - | Psat7g179200 | Unknown |
| - | - | - | - | Psat7g179240 | Unknown |
| - | - | - | - | Psat7g179280 | Transition metal ion binding |
| - | - | - | - | Psat7g179320 | DHHC palmitoyltransferase |
| - | - | - | - | Psat7g179360 | Aminotransferase class-III |
| - | - | - | - | Psat7g179400 | Pyridoxal phosphate-dependent transferase + major region + subdomain 1 |
| - | - | - | - | Psat7g179440 | Unknown |
| - | - | - | - | Psat7g179480 | Saccharopine dehydrogenase NADP binding domain |
| - | - | - | - | Psat7g179520 | TPR repeat region circular profile. |
| - | - | - | - | Psat7g179600 | Glycosyl hydrolases family 17 |
| - | - | - | - | Psat7g179640 | Glycosyl hydrolases family 17 |
| - | - | - | - | Psat7g179680 | Adaptin N terminal region |
| - | - | - | - | Psat7g179720 | Ubiquitin-conjugating enzyme |
| - | - | - | - | Psat7g179760 | DDT domain |
| - | - | - | - | Psat7g179800 | Domain of unknown function (DUF3594) |
| - | - | - | - | Psat7g179840 | Domain of unknown function (DUF3594) |
| S7LG7_449022566 | 7LG7 | 449022566 | Internode 2-3 | Psat7g223320 | Auxin response factor |
| - | - | - | - | Psat7g223360 | Acyl-CoA dehydrogenase + C-terminal domain |
| - | - | - | - | Psat7g223400 | Unknown |
| - | - | - | - | Psat7g223480 | MIZ/SP-RING zinc finger |
| - | - | - | - | Psat7g223440 | Cytochrome B561 + N terminal |
| - | - | - | - | Psat7g223560 | Proteasome subunit |
| - | - | - | - | Psat7g223520 | Unknown |
| - | - | - | - | Psat7g223600 | Histone H2B signature |
| - | - | - | - | Psat7g223680 | Oxidoreductase activity |
| - | - | - | - | Psat7g223760 | Ribosomal protein L9 + C-terminal domain |
| - | - | - | - | Psat7g223720 | Pheophorbide a oxygenase |
| - | - | - | - | Psat7g223800 | C2H2-type zinc finger |
| - | - | - | - | Psat7g223840 | Unknown |
| - | - | - | - | Psat7g223880 | Unknown |
| - | - | - | - | Psat7g223920 | Unknown |
| - | - | - | - | Psat7g223960 | Unknown |
| - | - | - | - | Psat7g224000 | Sec61beta family |
| - | - | - | - | Psat7g224040 | Voltage gated chloride channel |
